# Does a natural killer need a CAR?

**DOI:** 10.3389/fimmu.2025.1606126

**Published:** 2025-06-26

**Authors:** Kangbo Wang, Yumo Zhang, Zhibo Han, Youwei Wang

**Affiliations:** Institute of Medical Engineering & Translational Medicine, Tianjin University, Tianjin, China

**Keywords:** natural killer cells, chimeric antigen receptor, immunotherapy, CAR-NK cells, NK cell activation

## Abstract

Chimeric antigen receptor T cell (CAR-T) therapy has revolutionized cancer immunotherapy by overcoming intrinsic limitations in T cell activation, achieving remarkable success in treating relapsed or refractory hematologic malignancies in both pediatric and adult populations. Inspired by this success, researchers have turned their attention to natural killer (NK) cells—innate lymphocytes capable of recognizing and killing tumor cells without prior sensitization—and have begun designing CARs specifically adapted to NK cell biology. Unlike T cells, NK cells possess intrinsic cytotoxicity that does not solely rely on CAR-mediated activation. Moreover, NK cell-mediated antitumor responses can often be enhanced by simply increasing NK cell numbers, a strategy less effective in T cell-based therapies due to the complex mechanisms of tumor immune escape. This review explores the principles and strategies behind NK-adapted CAR design, critically examines whether CARs are essential for NK cell function, and discusses the opportunities and challenges in translating CAR-NK therapy into clinical practice.

## Introduction

Natural Killer (NK) cells are critical components of the innate immune system, recognized for their ability to identify and eliminate tumor cells without prior sensitization (1). However, in cancer patients, the functional capacity of NK cells is frequently compromised. In solid tumors, NK cells may fail to efficiently migrate to the tumor site, or, even if they successfully infiltrate the tumor microenvironment, they often exhibit impaired cytotoxic activity (2, 3). Emerging evidence also suggests that, under certain conditions, tumor-infiltrating NK cells may paradoxically contribute to tumor progression (4). In the case of hematological malignancies, NK cell dysfunction tends to be systemic, with global impairments in cytolytic function and increased expression of inhibitory receptors. These observations highlight a critical barrier to effective anti-tumor immunity and underscore the therapeutic potential of strategies aimed at restoring or enhancing NK cell responses. Approaches that increase NK cell abundance, improve their trafficking to tumor sites, or enhance their effector function represent promising avenues in the development of next-generation cancer immunotherapies.

Among the most prominent breakthroughs in current cancer immunotherapy is chimeric antigen receptor T (CAR-T) cell therapy. T cells are central components of the adaptive immune system, possessing the capability to recognize and eliminate tumor cells (5). However, their activation is a highly complex, multi-step process requiring precise coordination of antigen recognition, co-stimulation, and cytokine signaling (6, 7). This stringent activation threshold ensures immune precision while preventing unwanted autoimmunity (8). Tumor cells evade T cell surveillance by downregulating MHC molecules to reduce antigen presentation and detection, undergoing antigenic variation to selectively express poorly presented antigens, and expressing inhibitory ligands such as PD-L1, which bind to PD-1 on T cells, leading to exhaustion and functional impairment (9). CAR-T cell technology was developed to overcome the limitations of conventional T cell activation, particularly regarding antigen specificity and activation sustainability. By engineering T cells with CARs, CAR-T cell therapy enables direct tumor recognition and targeted activation, circumventing key mechanisms of tumor immune evasion (10). However, despite the remarkable progress in CAR-T cell therapy, researchers have increasingly recognized its inherent limitations. These include the necessity for autologous transplantation, prolonged manufacturing timelines, and the risk of cytokine release syndrome (CRS), among other challenges (11). Such constraints have hindered its broader clinical application, prompting investigations into alternative immune cell platforms with enhanced therapeutic potential.

Considering the cytotoxic similarities between NK cells and T cells, efforts to emulate the success of CAR-T therapy in NK cells led to the development of CAR-NK cells, and early studies in this field demonstrated that CARs endow NK cells with target-specific cytotoxicity (12, 13), marking a notable advancement in adoptive cellular immunotherapy.

Over the subsequent years, extensive preclinical studies validated the feasibility of CAR-NK cell therapy, establishing a critical foundation for its translation into clinical settings.

A CAR-NK clinical trial published in 2018 confirmed the safety profile of CAR-NK therapy in patients with relapsed/refractory acute myeloid leukemia (AML) but demonstrated no significant therapeutic efficacy, underscoring the need for further optimization of this approach (14). In the same year, Dan S. Kaufman and other researchers introduced a novel design paradigm: tailoring CAR structures specifically to align with the cytotoxic mechanisms of NK cells (15). Their experiments revealed that NK cell-based CAR constructs exhibited superior tumor suppression capabilities compared to conventional T cell-based CARs, significantly prolonging survival in tumor-bearing mice. This breakthrough provided a fundamental theoretical framework for subsequent advancements in CAR-NK engineering and functional refinement.

## NK cell-based CAR designs

**Figure 1:**
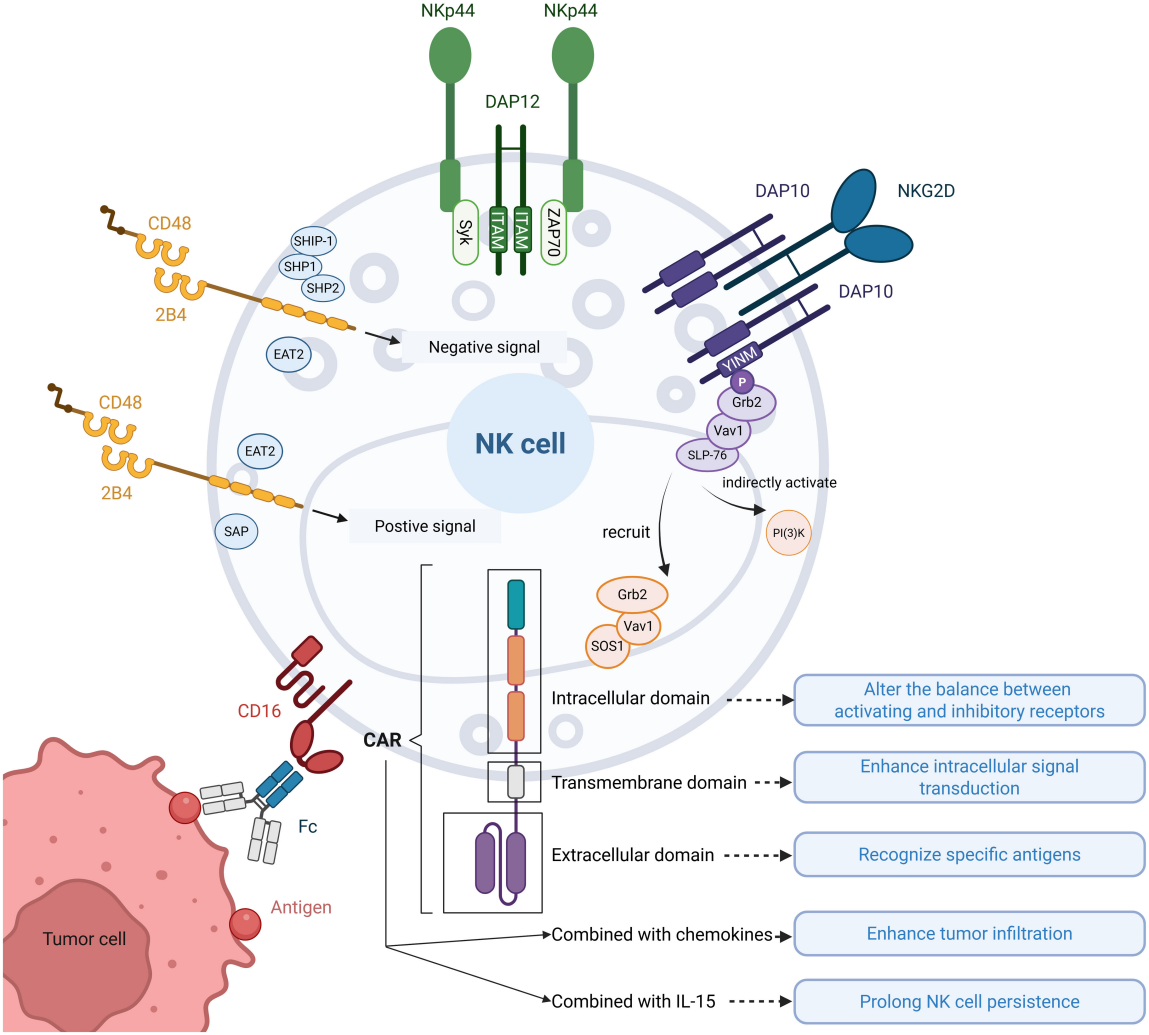
Illustration of NK cell activation and CAR-enhanced functionalities. Common activation pathways of NK cell receptors: ①DAP12 recruits Syk/ZAP70 through ITAM motifs carried in the cytoplasmic tail, which activates the downstream pathway and activates NK cells; ②NKG2D normally binds to DAP10 to form a hexamer, which recruits the Grb2-Vav1-SOS1 complex and indirectly activates PI(3)K through the YINM motif carried in the cytoplasmic tail of DAP10, triggering a downstream cascade of reactions to activate NK cells; ③Upon binding of 2B4 to its ligand CD48, tyrosine motifs in the cytoplasmic tail interact with SAP or EAT2 to transmit activating signals, and with SHIP-1, SHP1, SHP2, or EAT2 to transmit inhibitory signals; ④CD16 binds to the Fc segment of the antibody and mediates ADCC action to activate NK cells. CAR functions on NK cells: CAR modulate the receptor balance by altering activating/inhibitory signaling equilibrium, enhance intracellular signal transduction efficiency, and confer antigen-specific recognition. Furthermore, combinatorial strategies incorporating chemokines and IL-15 synergistically improve tumor infiltration and prolong NK cell persistence.

In the tumor microenvironment, NK cells face significant functional challenges, including dysregulation of inhibitory and activating receptor signaling and insufficient infiltration into solid tumors. CAR address these limitations by enabling targeted antigen recognition (16), rebalancing receptor-mediated immune signaling, enhancing tumor infiltration (17), and prolonging NK cell persistence (18). Moreover, CAR-NK cells address the unmet requirements of CAR-T therapy by offering potential for off-the-shelf product development, reduced side effects, and abundant cellular sources (19), thereby providing a foundation for improving NK cell-based immunotherapies. In this section, we summarized the strategies employed by researchers in designing CAR constructs based on NK cell activation mechanisms, while elucidating the functional enhancements conferred to NK cells through CAR engineering (**Figure 1**).

### DAP12

DAP12 is a type I transmembrane protein that is widely expressed in NK cells, myeloid cells and T cells (20, 21). DAP12 is a disulfide-linked homodimer that binds non-covalently to several activation receptors expressed on NK cells (e.g. KIR2DS, KIR3DS, NKG2C, Ly49D, NKp44, etc.). Its structure is divided into three parts, a short extracellular region containing two cysteines to mediate interchain disulfide bonding. A transmembrane structural domain containing two glutamate residues forming a localized negative charge that binds to lysine residues of the activated NK receptor by electrostatic attraction, thus facilitating the assembly of the receptor complex. An intracellular structural domain with a single ITAM motif induces cytokine production and cell-mediated cytotoxicity by recruiting the Syk/ZAP-70 family of kinases, triggering a downstream cascade of reactions (22). In the structure of NK-CAR, DAP12 can either act as an activation element alone or as a co-stimulatory signal together with other activation elements (e.g. CD3ζ, 2B4, 4-1BB, etc.) (23, 24). In addition, it has also been found that DAP12 has a role in stabilizing CAR expression on NK cells. Several *in vitro* experiments have found that DAP12 as an activation element is superior to CD3ζ for enhancing CAR-NK cells cytotoxicity (24, 25), although CD3ζ carries 3 ITAMs and DAP12 carries 1 ITAM. CAR-NK cells with DAP12 as an intracellular activation element targeting the NKG2DL has shown effects on reducing the number of tumor cells in the ascites of metastatic colorectal cancer patients and observed rapid tumor regression in the liver region (25).

### DAP10

DAP10, which is expressed in most myeloid, NK cells and CD8 T cells, is structurally very similar to DAP12, with a small extracellular region that also mediates disulfide bonding via cysteines to form homodimers (26). However, unlike DAP12, the signaling motif in the short cytoplasmic domain of DAP10 is a YINM sequence. Binding of DAP10 to the ligand promotes phosphorylation of the YINM motif, which in turn recruits the Grb2-Vav1-Sos1 complex and activates phosphatidylinositol-3-kinase(PI3K), triggering a downstream cascade of reactions that activate NK cells (27). NKG2D was the first receptor identified to bind to DAP10 (28). NKG2D is a type C lectin receptor consisting of two disulfide-linked type II transmembrane glycoproteins with short intracellular tails without signaling capacity. Therefore, to initiate signaling, human NKG2D needs to be coupled to the transmembrane adapter DAP10, which together form a hexameric structure assembled from one NKG2D homodimer and two DAP10 homodimers (29). Therefore, in the structure of NK-CAR, NKG2D often acts as a transmembrane structural domain and DAP10 as an intracellular structural domain (30–32). Both *in vitro* and *in vivo* experiments have demonstrated that this synergistic combination significantly enhances the anti-tumor effects of CAR-NK cells compared to those incorporating classical T cell-associated co-stimulatory domains such as CD28 or 4-1BB (15, 33, 34). Katayoun Rezvani and colleagues conducted an intriguing comparative analysis of NK-CAR constructs, demonstrating that CD28-based co-stimulatory domains outperformed DAP10, DAP12, and 4-1BB in functional assays (35). However, this study exclusively utilized CD28 as the transmembrane domain component. It’s unclear if switching to NKG2D transmembrane domains would maintain this efficacy hierarchy, as CD28 transmembrane domains appear to facilitate signal transduction specifically for CD28 co-stimulatory domains.

### NKG2D

NKG2D is a potent activating receptor that is constantly expressed on all NK cells, and as mentioned above, in NK cells, the intracellular tail of NKG2D is not signaling and requires binding to DAP10 for signaling. NKG2D-based CAR-NK cell therapy has demonstrated significant therapeutic potential in a variety of malignant tumors. NKG2D-based CARs mostly employ their extracellular recognition domains, while the combination strategies of transmembrane region and intracellular co-stimulatory structural domains show diversification, among which 4-1BB is the more commonly used co-stimulatory structural domain (15, 25, 36–38). In addition to being part of the CAR structure, the researchers developed a soluble CAR molecule based on the NKG2D-NKG2DL axis. This molecule exists as a free protein, and by simultaneously recognizing specific antigens on the surface of tumor cells (e.g., CD20) and NKG2D receptors on the surface of NK cells, this soluble CAR triggers NK cell activation by means of a bridging mechanism, which ultimately achieves the killing effect on tumor cells (39).

### 2B4

2B4, also known as CD244, is expressed in all NK cells as well as CD8 αβ T cells, γδ T cells, basophils, and monocyte subpopulations (40). 2B4 has a well-defined activation function (e.g., triggering of lysis activity, proliferation, and cytokine secretion) in NK cells, whereas in T cells and myeloid cells, its antibody cross-linking did not induce significant effector functions (41). Human 2B4 has an N-terminal extracellular structural domain, a variable Ig-like structural domain, a constant type II Ig-like structural domain, a single transmembrane structural domain, and a cytoplasmic tail containing a tyrosine motif (42). In NK cells, 2B4 binds CD48 with high affinity. The intracellular tyrosine motif interacts with SAP to transmit activating signals, with SH2 phosphatases (SHP1, SHP2, and SHIP-1) to transmit inhibitory signals, and with EAT2 to transmit either activating or inhibitory signals (43). 2B4 can effectively co-stimulate the signaling of other activating NK cell receptors (44), In the structure of NK-CAR, 2B4 is often used as a co-stimulatory structural domain with DAP10, DNAM1, etc., and seldom as an activation element alone. It has been demonstrated that 2B4 as a co-stimulatory domain significantly enhances the cytotoxicity of CAR-NK cells on target cells compared to classical CARs designed for T cells (with CD28 or 4-1BB as co-stimulatory domains, etc.), both for hematological tumors and for solid tumors (15, 30–32, 34, 45). When used as a co-stimulatory structural domain, 2B4 itself is often utilized as a transmembrane structural domain. This is because it has been shown that if the intracellular region of CD28 is used in a 2B4-based CAR structure, the activation of 2B4 will be less effective than that of CD28 (46). Despite the excellent performance of 2B4 as a CAR co-stimulatory structure in *in vitro* experiments, the improvement of disease in *in vivo* tumor models was rather limited (32). This suggests that the design of 2B4 for CAR-NK cells needs to be adapted more based on *in vivo* experiments.

### CD16

CD16 is an Ig superfamily molecule involved in antibody-dependent cell-mediated cytotoxicity (ADCC) and is expressed at high levels in the CD56(dim) NK cell subset (47). CD16 is the only activation receptor for which a single activation receptor is sufficient to activate natural cytotoxicity without the need for complementary combinations of other activation receptors (48) Therefore, in the design of CAR-NK cells, CD16 serves as both a transmembrane and intracellular structural domain without the need for other activation elements (49). In addition to being part of the CAR structure, CD16 is more often co-expressed with CAR to mediate or enhance ADCC action (50–52). The application of CAR-NK cells designed based on CD16 has been expanded to multiple cancer types, covering hematological malignancies (e.g., acute myeloid leukemia, lymphomas) and solid tumors (e.g., renal cell carcinoma, breast cancer, lung adenocarcinoma). The role of CD16 has been validated in both *in vitro* experiments and in tumor-bearing mice (49–52). However, as of this writing, no CD16-based CAR-NK cell technology has entered the clinical trial stage and publicized relevant research results.

### Cytokines

The cytokines utilized in CAR-NK cell therapy can be categorized into two major classes: interleukins and chemokines. IL-15 has emerged as the most extensively utilized interleukin in CAR-NK cell research, which plays a critical role in regulating NK cell survival, maturation, proliferation, and persistence. Depending on the state of NK cells and infection conditions, IL-15 binding to IL-15R on NK cells induces three pathways, namely, the Ras-Raf-MAP kinase, STAT5, and mTOR pathways (53). In NK-CAR, the cytotoxicity of NK cells is often further enhanced by ectopic expression of IL-15 (46). It has been shown that although CAR can enhance the killing ability of NK cells, this benefit is transient, and thus the introduction of IL-15 is needed to enhance the persistence of CAR-NK cells (32). Furthermore, studies have demonstrated that NK cells experience rapid attenuation of effector functions following infiltration into solid tumor microenvironments. Administration of IL-15 has been shown to effectively counteract this functional exhaustion and enhancing tumor control efficacy (3). This suggests that IL-15 may constitute a promising strategy to address the challenges in solid tumor immunotherapy. In clinical trials, IL-15 significantly increased the number of circulating NK cells (54). Therefore, some of the clinical trials of CAR-NK cells conducted so far have co-expressed IL-15 along with CAR expression to make the activity of NK cells more persistent, and these experiments have mostly focused on tumors in the CD19^+^ blood system (55, 56). Chemokines play a critical role in enhancing the infiltration capacity of CAR-NK cells into solid tumors. Current studies have demonstrated that enhancing NK cell expression of tumor-associated chemokines through electroporation (57) or lentiviral transduction (58), when combined with CAR engineering, significantly improves tumor homing capabilities. A recent study further demonstrated that chemical pretreatment of NK cells could achieve comparable homing enhancement through a technically simpler and more expedient approach, requiring only the administration of a specialized medium to tumor-bearing mice models (59). This strategy presents notable advantages in clinical translatability.

## *In vivo* persistence of CAR-T vs. CAR-NK cells

In the realm of CAR therapies, the *in vivo* persistence of modified cells is a critical determinant of therapeutic efficacy. CAR-T cells are predominantly derived from autologous T cells, mitigating the risk of immune rejection and allowing these modified cells to persist long-term within the host. Clinical observations have documented CAR-T cell persistence extending for years post-infusion, contributing to sustained antitumor activity (60). Another notable advantage of autologous CAR-T cell therapy is the potential development of memory T cells, which provide long-lasting immunity by continuously surveilling and eliminating tumor antigen-expressing cells. The generation of memory CAR-T cells has been associated with improved persistence and durable therapeutic responses (61).

CAR-NK cells can be sourced from various origins, including autologous peripheral blood NK cells, umbilical cord blood-derived NK cells, induced pluripotent stem cells (iPSCs), and the NK-92 cell line (15, 56, 62, 63). Each source presents distinct advantages and limitations concerning cell expansion, genetic modification, safety, and clinical applicability. Allogeneic NK cells, sourced from umbilical cord blood or iPSC-derived NK cells, provide a readily available and healthy cell population. However, their persistence *in vivo* is generally limited, and they may not endure long enough to achieve sustained antitumor activity comparable to CAR-T cells. Induced pluripotent stem cell derived natural killer cells can be produced in large quantities and are amenable to genetic modifications. Theoretically, it is possible to generate autologous iPSC-derived NK cells; however, the production process for such cells is complex and costly, which may limit widespread clinical application. Currently, there are no reports of autologous iPSC-derived CAR-NK cells being used in clinical settings. The NK-92 cell line, initially established from peripheral blood mononuclear cells of a patient with non-Hodgkin lymphoma (64), demonstrates consistent scalability under Good Manufacturing Practice (GMP) conditions (65), providing a robust platform for allogeneic “off-the-shelf” cell therapy development. However, its clinical application requires mandatory pre-infusion irradiation to abrogate *in vivo* proliferative capacity and tumorigenic potential. While CAR-modified NK-92 therapies have shown safety in clinical trials, the limited therapeutic efficacy may correlate with irradiation-induced functional deficits in the engineered cells (14).

To minimize immune rejection and enhance persistence, autologous peripheral blood-derived NK cells can be utilized for CAR-NK cell therapies. However, primary NK cells exhibit limited ex vivo expansion capacity and low transduction efficiency, which may impede their therapeutic potential (66). Additionally, unlike T cells, NK cells typically do not form long-lived memory cells, potentially limiting the duration of their antitumor effects in patients. Therefore, in clinical practice, autologous NK cells are rarely used to prepare CAR-NK cells either. Enhancing the efficiency of genetic modifications in NK cells or reducing the production costs of iPSC-derived NK cells are pivotal technological advancements necessary to promote the clinical translation of CAR-NK cell therapies.

## NK cell quantity in immunotherapy

CAR-T cells have demonstrated significant cytotoxicity against tumor cells that are inherently resistant to conventional T-cell-mediated killing. In contrast, CAR-NK cells primarily target tumor cells that are already sensitive to NK cells, with CAR modification further enhancing their cytotoxic potential (15). Notably, the enhanced cytotoxicity observed in CAR-NK cells may also be achieved by optimizing the activity and quantity of non-genetically modified NK cells. A common phenomenon observed in preclinical studies of CAR-NK cells is that NK cell-mediated cytotoxicity against tumor cells, both *in vitro* and *in vivo*, increases with the effector-to-target ratio. This suggests that the therapeutic efficacy of CAR-NK cells can, to some extent, be augmented by increasing the quantity of NK cells. Therefore, further investigation is needed to assess whether CAR modification can confer NK cells with an enhanced antitumor effect comparable to that of CAR-T cells, as well as to evaluate their safety and unique advantages in clinical applications. Some studies have reported that CAR-NK cells do not exhibit cytotoxicity against normal cells that do not express the target antigen but may attack normal cells that do express tumor antigens (24). The clinical relevance of this phenomenon may arise in cases where tumor cells lose part or all of the danger signals required for NK cell activation while still retaining the CAR-targeted antigen. Whether this scenario is commonly observed in tumor progression and development remains an open question for further research. Moreover, this observation appears to contradict the immune tolerance mechanisms of NK cells, which typically do not target self-cells, highlighting a potential safety concern that warrants further exploration.

Evidence from current clinical studies shows that CAR-NK cell trials often involve small populations and a broad range of doses, with the minimum reported dose reaching as low as 1×10^5^ cells/kg (56). Notably, some clinical studies have used non-genetically modified NK cells at doses up to 3×10^8^ cells/kg, with no significant toxicity observed (67). Existing clinical data suggest that the efficacy of CAR-NK cells is comparable to that of ex vivo-expanded NK cells, with no conclusive evidence indicating superior clinical outcomes for CAR-NK cells over their unmodified counterparts. This may be due to the absence of direct side-by-side comparisons in clinical trials. Conducting a comparative study within the same clinical trial to directly evaluate the therapeutic effects of NK cells versus CAR-NK cells is challenging. Thus, only through large-scale clinical studies and cumulative data analysis can the differences in efficacy between CAR-NK cells and unmodified NK cells in tumor immunotherapy be accurately determined. However, these differences may not be as pronounced as those observed between CAR-T cells and unmodified T cells.

Since high-dose NK cell administration has not been associated with significant toxicity in clinical studies, a key consideration is whether sufficient NK cells can be generated to meet therapeutic demands. Currently, NK cells derived from adult peripheral blood can be expanded up to approximately forty-thousand-fold ex vivo (68). Umbilical cord blood-derived NK cells can be expanded using two different approaches: direct expansion of NK cells from umbilical cord blood, achieving an approximate two-thousand-fold increase (69), or differentiation of CD34^+^ hematopoietic stem cells from umbilical cord blood into NK cells, followed by expansion, resulting in an approximately fifteen-thousand-fold increase (70). Furthermore, induced pluripotent stem cell (iPSC)-derived NK cells, theoretically, possess unlimited proliferative potential, offering a promising source for large-scale clinical applications. It is worth noting that CAR-NK cells derived from a single unit of cord blood have already demonstrated anti-tumor effects in clinical applications (56). Therefore, to fairly present the genetic editing and expansion of NK cells, enhancing NK cell function through genetic engineering and improving expansion efficiency to achieve higher therapeutic doses should both be considered important areas of investigation in NK cell-based cancer immunotherapy.

## Conclusion

NK cells and T cells share remarkable similarity in how they execute tumor cell killing once activated. However, the mechanisms governing their activation are fundamentally distinct. Does this difference carry biological significance, or can the success of CAR-T cells be simply transplanted onto NK cells? Rather than directly applying T cell-based strategies, it may be time to ask whether NK cells require solutions uniquely suited to their own biology.
